# Reconstructive flap surgery in head and neck cancer patients: an interdisciplinary view of the challenges encountered by radiation oncologists in postoperative radiotherapy

**DOI:** 10.3389/fonc.2024.1379861

**Published:** 2024-04-11

**Authors:** Juliette Thariat, Florent Carsuzaa, Arnaud Beddok, Sophie Deneuve, Pierre-Yves Marcy, Anna Merlotti, Catherine Dejean, Bernard Devauchelle

**Affiliations:** ^1^ Department of Radiotherapy, Centre François-Baclesse, Caen, France; ^2^ Corpuscular Physics Laboratory, IN2P3, Ensicaen, CNRS UMR 6534, Caen, France; ^3^ Faculté de Médecine de Caen, Université de Normandie, Caen, France; ^4^ Department of Otorhinolaryngology - Head and Neck Surgery, University Hospital of Poitiers, Poitiers, France; ^5^ Institut Curie, PSL Research University, University Paris Saclay, Inserm LITO, Orsay, France; ^6^ Gordon Center for Medical Imaging, Massachusetts General Hospital, Harvard Medical School, Boston, MA, United States; ^7^ Surgical Oncology Department, Centre Léon Bérard, UNICANCER, Lyon, France; ^8^ Inserm, U1296 Unit, “Radiation: Defense, Health and Environment”, Centre Léon Bérard, Lyon, France; ^9^ Polyclinics ELSAN Group, Department of Radiodiagnostics and Interventional Imaging, PolyClinics Les Fleurs, Ollioules, France; ^10^ Radiotherapy Department, S. Croce & Carle Teaching Hospital, Cuneo, Italy; ^11^ Radioophysics, Centre Lacassagne, Nice, France; ^12^ Departement of Maxillofacial Surgery, University Hospital of Amiens Picardy, Research Unit, UR7516 CHIMERE, University of Picardy Jules Verne, Institut Faire Faces, Amiens, France

**Keywords:** head and neck cancer, reconstructive surgery/flap, radiotherapy, delineation, target volumes, relapse, functional outcomes, guidelines

## Abstract

**Background:**

Major advances have been made in reconstructive surgery in the last decades to reduce morbidity in head and neck cancer. Flaps are now present in 80% of patients with oral cavity cancer to cover anatomic, functional, and cosmetic needs. However, gaps in interdisciplinary innovation transfer from surgery to postoperative radiotherapy (poRT) remain challenging. We aimed to provide an interdisciplinary view of the challenges encountered by radiation oncologists in planning head and neck postoperative radiotherapy.

**Methods:**

A systematic and critical review was conducted to address areas of optimization in surgery and radiology that may be relevant to poRT.

**Results:**

Despite extensive surgical literature on flap techniques and salvage surgery, 13 retrospective series were identified, where flap outcomes were indirectly compared between surgery alone or poRT. These low-evidence studies suggest that radiotherapy accelerates flap atrophy, fibrosis, and osteoradionecrosis and deteriorates functional outcomes. Preliminary evidence suggests that tumor spread occurs at the flap–tissue junction rather than in the flaps. One prospective 15-patient study showed 31.3% vs. 39.2% flap volume reduction without or with poRT. In an international consensus, experts recognized the needs for optimized flap-sparing poRT against flap-related functional deterioration and bone damage. CT, MRI, and PET-CT modalities show potential for the delineation of the junction area between native tissues and flap for flap segmentation and to characterize flap-specific changes quantitatively and correlate them with patterns of relapse or complications.

**Conclusion:**

Flap management in poRT is insufficiently documented, but poRT seems to damage flaps. Current gaps in knowledge underscore the need for prospective flap assessment and interdisciplinary trials investigating flap morbidity minimization by flap-sparing poRT planning.

## 1 Introduction

In the last two decades, substantial improvements have been made in surgery, radiotherapy, systemic treatments, and supportive care of head and neck cancer (HNC). Over 75% of HNC patients have advanced stages at diagnosis and elect for combined treatments (1). Often inevitable to maximize the chance of cure, combined treatments are also responsible for cumulative late toxicities (2). Surgery is the mainstay of treatment, with radiotherapy advocated in about 60% of patients in the definitive (chemo)radiotherapy or postoperative (poRT) setting (3). Primary tumor resection is increasingly performed using reconstructive surgery and mini-invasive/mini-morbid approaches, with de-escalation when appropriate (4, 5). Reconstructive surgery aims to mitigate the effects of tumor resection and is standard practice in many cancer sites (6) to restore the anatomy and function. Flaps, i.e., vascularized autologous tissues repairing a tissue defect or grafts protecting or sustaining organs (7) and dental implants, fixating plates, and prosthetic materials, are frequent practice, yet interdisciplinary transfer of high-end technology suffers from gaps in knowledge at interfaces between disciplines. The choice of the reconstructive technique and materials depends on patient comorbidities, tumor bulk and site, and surgical expertise (8–10). Reconstructive surgery is no longer limited to surgical salvage in case of tumor relapse in irradiated tissues with a flap to protect the carotid artery from salivary leakage.

As a result, flaps are now present in over 80% in oral cavity cancer poRT series and 50% of all tumor sites (11, 12). Flaps can allow the anticipation of more generous margins (13, 14). The next challenge is high-end surgical technology transfer to the poRT planning to refine target volumes in the presence of a flap to retain full functionality. This critical review addresses the multidisciplinary issues of the management of flaps in HNC poRT planning and poRT outcomes.

## 2 Search strategy and selection criteria

PubMed was searched using the terms: (flap[Title]) AND (radiotherapy[Title]) for the 2008–2023 period. Free flaps do not hold the same radiotherapy planning challenges as local flaps on one hand or pedicled flaps on the other hand; all flaps were therefore included. The initial sifts focused on HNC. Further eligibility criteria excluded articles of salvage reconstructive surgery in irradiated areas and case reports (**Supplementary Figure S1**: flowchart). This search process was extended to comprehensively address aspects pertaining to flap reconstruction and poRT in HNC with articles sourced from the reference lists of the primary articles. It enabled us to cover surgical techniques, imaging strategies, and radiation toxicity. The final set of articles was reviewed, providing a comprehensive perspective on the complex interplay between flap reconstruction and poRT in HNC patients.

## 3 Surgical management of flaps

### 3.1 Flap reconstructive surgery

Primary closure and spontaneous healing may be sufficient for small tumors. In many other cases, curative resection requires a wide excision to obtain safe margins and for the subsequent functional repair of head and neck anatomy. Grafts are mostly made of non-vascularized fascia or skin and exert a mechanical role rather than restore a function. They can cover variably large surfaces of defect, such as the fascia lata graft to protect the dura during sinonasal surgery. Made of autologous tissues having their own vascular supply, flaps are often more appropriate than grafts to fulfil the functional needs associated with a volumetric substance loss (15). Flaps are transposed geometrically with their blood supply to the tissue resection site, the “tumor bed”, from a loco-regional non-tumoral area. Exploiting advances in microvascular surgery, free flaps are harvested from distant donor body parts, with vessels micro-anastomosed between the donor and recipient sites. Such flaps are expected to be versatile to contribute to vital functions with high fidelity. Chimeric flaps associate soft and bony tissues. In bone flaps, hardware, i.e., plaques and screws made of metal (or artefact-free carbon fiber), fix the bone flap to the native bone anatomy (16). In uncertain cases, however, reconstruction may be delayed after definitive negative margins are obtained. Anticipating flap reconstruction may allow for larger resections and may improve local control and progression-free survival (17). It also allows optimal flap choice based on the estimates of extent of the tissue defect clinically and by conventional preoperative imaging using CT or MRI of the head and neck. Preoperative assessments of comorbidities and vascular supply quality are also needed (18).

### 3.2 Flap types

The types of flaps include: (a) the local flap, with geometric reshaping/positioning to adjacent tissue; (b) the regional flap, with rotation of the flap vascular system, and (c) the distant free flap with transfer of microanastomosed tissue. Flaps can consist of one tissue type, such as cutaneous flaps, or, more frequently, several tissue types such as myocutaneous, fasciocutaneous, and bone flaps; the latter also often have a soft tissue part. Flaps vary depending on the complexity of the defect (19). The native tissue is not necessarily replaced by the same tissue, and the native mucosae is often substituted for skin from the donor site. Flap reconstruction is continuously evolving toward the use of more versatile flaps (agile to comply with functional requirements), including composite/chimeric (made of several tissue types), multiple (made of different flaps such as, for example, the fibula flap and the anterolateral thigh flap, transplanted sequentially during the same surgery, to both get bone and enough skin volume at the recipient site), and “free style” (highly customized operator dependent) flaps to cover unusual needs. Technical advances and new flaps are variably integrated by surgical teams in time.

### 3.3 Basic principles of flap selection

When the primary closure of a relatively small defect is not feasible and surgical margins are negative, local flap reconstruction is usually the preferred procedure. The benefits include ease of harvest, short operating room time with little patient morbidity constraints, short hospital length of stay, and low morbidity at the donor site. The drawbacks include limited size, risk for partial necrosis, or vascular pedicle damage during tumor resection and wound dehiscence. The facial artery myomucosal (FAMM) and buccinator local flaps can, as an example, reconstruct a small floor of mouth tumor.

**Figure 1:**
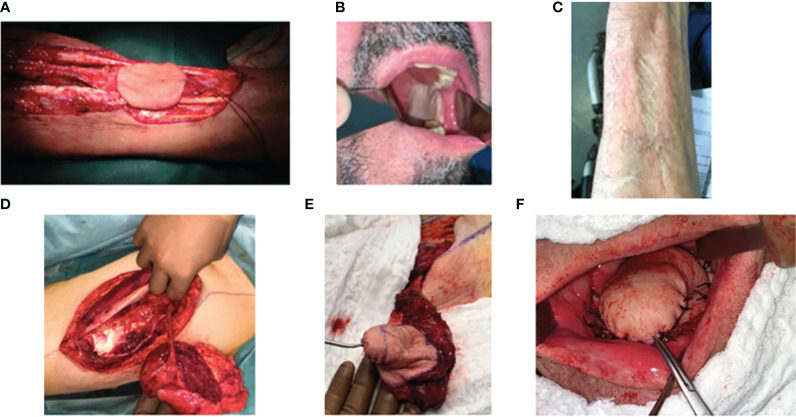
Surgical harvest and use of radial forearm and anterolateral thigh free flaps for reconstructive surgery. **(A, B)** Radial forearm free flap for the reconstruction of a retromolar trigone tumor. **(A)** Harvesting of the flap pedicle from the radial artery and vein, **(B)** flap positioning in the oral cavity, and **(C)** donor site. (**D**–**F**) Anterolateral thigh flap for the reconstruction of a mobile tongue tumor. **(D)** Harvesting of the flap pedicle on the peroneal artery, **(E)** flap during the modeling step, and **(F)** flap positioned in the oral cavity.

Free flaps can be larger than local flaps and are more versatile. A free flap is chosen based on the size of the surgical defect and donor tissue bulk availability, need for bone or soft tissue only, thickness, and pliability along with mobility of the restored organ (such as the tongue). Surgical oncology with free flap reconstruction requires successive operative steps, e.g., tumor resection, flap harvesting at the donor site, flap transplantation to the recipient site, and therefore prolonged general anesthesia, which may be contraindicated in case of severe comorbidities. Morbidity of harvesting at the donor site is also considered, and feasibility of vascular microsurgery is dependent on the quality of both recipient and donor arteries, which may be inadequate (atherosclerosis) or depleted (previous local treatments). Common free flaps include (but are not limited to) the radial forearm, anterior lateral thigh, fibula, iliac, scapula, latissimus dorsi (in particular when team expertise with perforator flaps is limited), and jejunal free flaps (**Table 1**). The radial forearm flap, for example, may be used for reconstructions of the oral cavity, tongue, palate, nose, face, scalp, lip, and pharynx (20) (**Figure 1**). The anterolateral thigh flap can also be used for larger and thicker substance loss. The fibula flap may be used to reconstruct the upper maxillary or mandibular bone (20). Osteotomies can be prepared preoperatively with 3D planning software and cutting guides, while soft tissue components are usually shaped peri-operatively. The jejunal flap may be used for circumferential pharyngeal and esophageal defects (20). When local flaps do not adequately fulfill the needs and free flaps are not feasible, pedicle flaps, such as the pectoralis major flap, may be used. This is particularly the case in patients with severe comorbidities or after a first flap failure.

**Table 1:** Main types of flaps and their source artery.

Type of flap	Flap	Location of defect	Source artery	Advantage	Limitation
Local flap	FAMM	OC, OPH	Facial artery	Ease of harvesting, minimal donor site morbidity, constant vascularization	Limited by the laxity of the buccal mucosa for direct closure
	Buccinator	OC, OPH	Buccinator artery	Ease of harvesting, minimal donor site morbidity, can be used in case of ligation of the facial artery	Small size
	Submental flap	OC, OPH	Submental branch of the facial artery	Ease of harvesting, minimal donor site morbidity	Not during concurrent neck dissection, unless preservation of the submandibular branch of the facial artery (and venous drainage) is feasible, as it may potentially reduce the risk of osteoradionecrosis
Regional flap	Pectoralis major myo-cutaneous flap	OC, OPH, HPL	Thoraco-acromial artery	Ease of harvesting, reliable vascularization	Limited reach, neck contracture due to fibrosis, bulge of the neck
	Latissimus dorsi	OC, OPH, HPL	Thoraco-dorsal artery	Ease of harvesting, reliable vascularization, and larger size compared to the pectoralis major flap; can be utilized as a free flap for reconstruction purposes	Limited reach, neck contracture due to fibrosis, bulge of the neck
	Supraclavicular artery island flap	OC, OPH, HPL	Supra-clavicular artery	Thickness and pliability	Potential vascularization issues leading to distal necrosis, the possibility of an unsightly scar, and persistent sensitivity or altered innervation
	Internal mammary artery flap	HPL	Internal mammary flap	Thin, pliable tissue; wide arc of rotation; perforator flap	
Free flap	Radial forearm fasciocutaneous free flap	OC, OPH, HPL, maxillary bone	Radial artery	Thickness and pliability	Unfavorable donor site appearance, risk of tendon exposure due to delayed healing, ligation of a major vascular axis of the arm, potential complications affecting the aesthetic outcome and healing process
	ALT	OC, OPH, HPL, maxillary bone	Descending branch of lateral femoral circumflex artery	Volume variability, minimal donor site morbidity perforator flap (allows thinning/highly customized shaping)	Less pliable than radial forearm free flap
	Fibula free flap (for bone defects)	HPL, maxillary bone	Fibular artery	Length of bone available, minimal donor site morbidity	Frailer bone vascularization with an increasing number of osteotomies; arterial supply from the inferior limbs may be frail in tobacco-smoking patients
	Scapular free flap (for bone defect)	HPL, maxillary bone	Circumflex scapular artery	Robust vascularization, similar structure to the mandibular angle; size of vessels, similar structure as the mandibular angle; many possibilities of association with soft tissue flaps (such as latissimus dorsi)	Patient position may need to be changed during surgery (supine with table rotation); donor site morbidity (cosmesis, movements)
	Iliac flap	OC, OPH	Deep circumflex iliac artery	Rich vascular supply of bone, better implant osteointegration	Short pedicle
	Jejunal free flap	HPL	Superior mesenteric artery	Robust vascularization, less salivary fistula	Donor site morbidity, poor-quality esophageal voice

FAMM, facial artery musculo-mucosal flap; ALT, anterolateral thigh free flap; OC, oral cavity; OPH, oropharynx; HPL, hypopharynx/larynx; maxillary bone, maxillary bone for facial reconstruction.

Although there is no consensual algorithm for the choice of a flap among many flaps (**Table 1**), the features that should constitute an ideal flap for head and neck soft tissue reconstruction may be summarized as follows: (1) the tissue harvested should be pliable so as to not impair movement and function in its recipient position in the head and neck, (2) the pedicle should be long, large, and consistent between source and recipient, (3) small and large flaps with variable thickness should be possible, (4) harvesting the flap and resecting the tumor should be feasible in the same position (usually supine), (5) the donor site morbidity should be low, both in terms of function and cosmesis, and (6) there should be sensitive nerves available with limited morbidity (21)—for example, the radial forearm was defined in the 1980s and is commonly used but may become less popular due to aesthetic donor site morbidity. For that reason, and for its larger size and versatility, the anterolateral thigh flap is also common. Perforator flaps are used since the 2000s and may be considered for their rich vascular supply. In bone reconstruction, the fibular flap is often considered to reconstruct mandibular defects (22). One might also consider the scapula in case of atherosclerosis of the inferior limbs or the iliac flap for its favorable curvature and shape of the bony part as well as muscular bulk to fill cavities to provide intraoral lining (23, 24).

It is beyond the scope of this article to standardize the choice between flap types (25) and perioperative regimens or the reporting of complications of patients undergoing free flap reconstructive surgery (26–28).

### 3.4 Vascular supply of flaps

The vascular supply of flaps originates directly from the native tissue artery in case of local (island artery) and regional/pedicled (axial artery) flaps. Free flaps rely on a transected reconnected source/recipient artery and vein. In the case of perforating flaps, harvesting includes a time of dissection of the perforating vessels from donor tissues, such as muscle, which will thus be left in place, preserving the function of the donor site. Perforating vessels originate in the source axial vessels, pass through tissues, besides interstitial connective tissue and fat, and allow the flap vascularization and thus shaping (such as thinning to only use the fatty and skin components of a thigh flap, for example) (29). The vascularization of the flap becomes progressively autonomous from that provided by its pedicle thanks to the angiogenesis of distal vascular branches formed from the surrounding recipient tissue.

### 3.5 Toward high-fidelity flap reconstruction

Reconstruction surgery is in constant progress to improve flap management and aesthetic/functional outcomes.

#### 3.5.1 Flap innervation

While flap vascularization is critical to flap vitality, flap innervation has pros and cons (30, 31). Motor nerve innervation (conservation or transection–reconnection) could limit muscle atrophy (further to fatty involution of inactive muscle) but should avoid dysesthestic and uncomfortable aberrant contractions (32). Therefore, the nerves of pedicled flaps (such as the latissimus dorsi) are often transected (33), and nerve graft is rarely performed in free flaps. Sensitive innervation should achieve superior sensitive recovery in innervated than non-innervated flaps, which may translate in better swallowing and speech function (34) and better quality of life (29). It has also been suggested that bone flap innervation might promote bone regeneration and turnover and subsequently less bone resorption (35). The assessment of the benefits of sensitive innervation warrants further matched or randomized studies.

#### 3.5.2 Overcompensation of soft tissue flap volume

Anticipating spontaneous flap atrophy over time, it is common surgical practice to overestimate the flap volume 1.6 times larger than the intraoperative defect resulting from the tumor resection (36). In the short term, patients are informed that they may experience transient difficulties swallowing and speaking in the first 6 months. Overcompensation of flap volume 20%–30% larger than the actual defects is often intuitively and empirically (with little inter-surgeons’ reproducibility) performed, yet with limited tools to predict ultimate flap volume (36). Some surgeons recommend to maintain body weight (37, 38) and use higher fat-to-muscle or no muscle (such as perforator flaps) (37–39). Skin atrophy is another possible evolution of flaps. Overcompensation is even more common in anticipation of poRT and has significant implications for poRT planning (see Section 5).

#### 3.5.3 Bone flap shaping

Functional restoration following segmental mandibulectomy or maxillectomy remains challenging despite substantial technology advances. Instead of a bone flap, a soft tissue flap is occasionally associated with a reconstruction plate in patients unfit for bone reconstruction (particularly for anterior lesions). In a meta-analysis of 2,379 patients undergoing reconstruction using a soft tissue flap with a plate, the risk of plate fracture was 5% and of extrusion 20%, while after bone reconstruction, the risk of extrusion was 10%. Bone reconstruction is also expected to be more functional (40). Bone flaps may rely on the use of bone, soft tissues, and a metallic (usually titanium) reconstruction bridging plate. However, plate exposure, osteitis, and osteoradionecrosis often exceed 30% in the long term; alternate options to fixation plates have been evaluated. Miniplates may reduce the risks and be more appropriate for patients who need poRT (41), yet in anticipation of poRT planning and complications, unmet needs remain in the engineering of plates with non-metallic carbon fiber materials that would support hard mechanical constraints and avoid delineation and dose uncertainties.

High-end technology in the field of bone reconstruction is currently dominated by the purpose of high-fidelity bone shaping. Virtual surgical planning, 3D-printed osteotomy guides, and preoperatively bent or custom-milled mandibular reconstruction plates have been shown to ease intraoperative decision making and reduce operative time. With such a purpose in mind, the selection criteria for bone flaps include the possibility of multiple osteotomies while keeping in mind to not impair vascularization, to resist mechanical constraints, to harbor dental implants. The early promising results of such technologies should be confirmed in the mid/long term (42)—for example, the high number of osteotomies of hyperconformal mandibular reconstruction could, in theory, be associated with a substantial damage to the periosteum, a factor that might result in frail vascularization and a higher risk of osteoradionecrosis.

#### 3.5.4 Dental rehabilitation

Common maxillary and mandibular composite free flaps may be used for dental implants (43). A systematic review of 2,626 implants placed into fibula, iliac crest, scapula, and radial forearm free flaps revealed a pooled 5-year survival rate of 94% of implants in the fibula and iliac crest. Factors affecting dental implant survival included implantation after poRT (HR 2.3, 95% CI: 1.2–4.6, *P* = 0.027). Immediate dental implantation has been advocated to promote more efficient rehabilitation, better patient acceptance, and aesthetics. According to a meta-analysis including randomized trials, immediate loading may, however, result in a higher incidence of implant failure compared to delayed loading (43). Immediate dental implants do not seem to increase the risks of osteonecrosis and osteoradionecrosis. However, case selection and publication biases seem to exist and should address the total number of immediate dental implants over the total number of HNC patients requiring dental implants with bone reconstruction. Another recent meta-analysis suggested that implants should be placed in the native bone rather than in bone grafts in case of poRT (44). Controversial issues remain in dental rehabilitation in the context of radiotherapy.

## 4 Flap changes and their clinical and radiological assessment

Flaps may be distinguished on imaging from native tissues based on their specific tissue components and anatomic asymmetry.

### 4.1 Imaging characteristics

**Figure 2:**
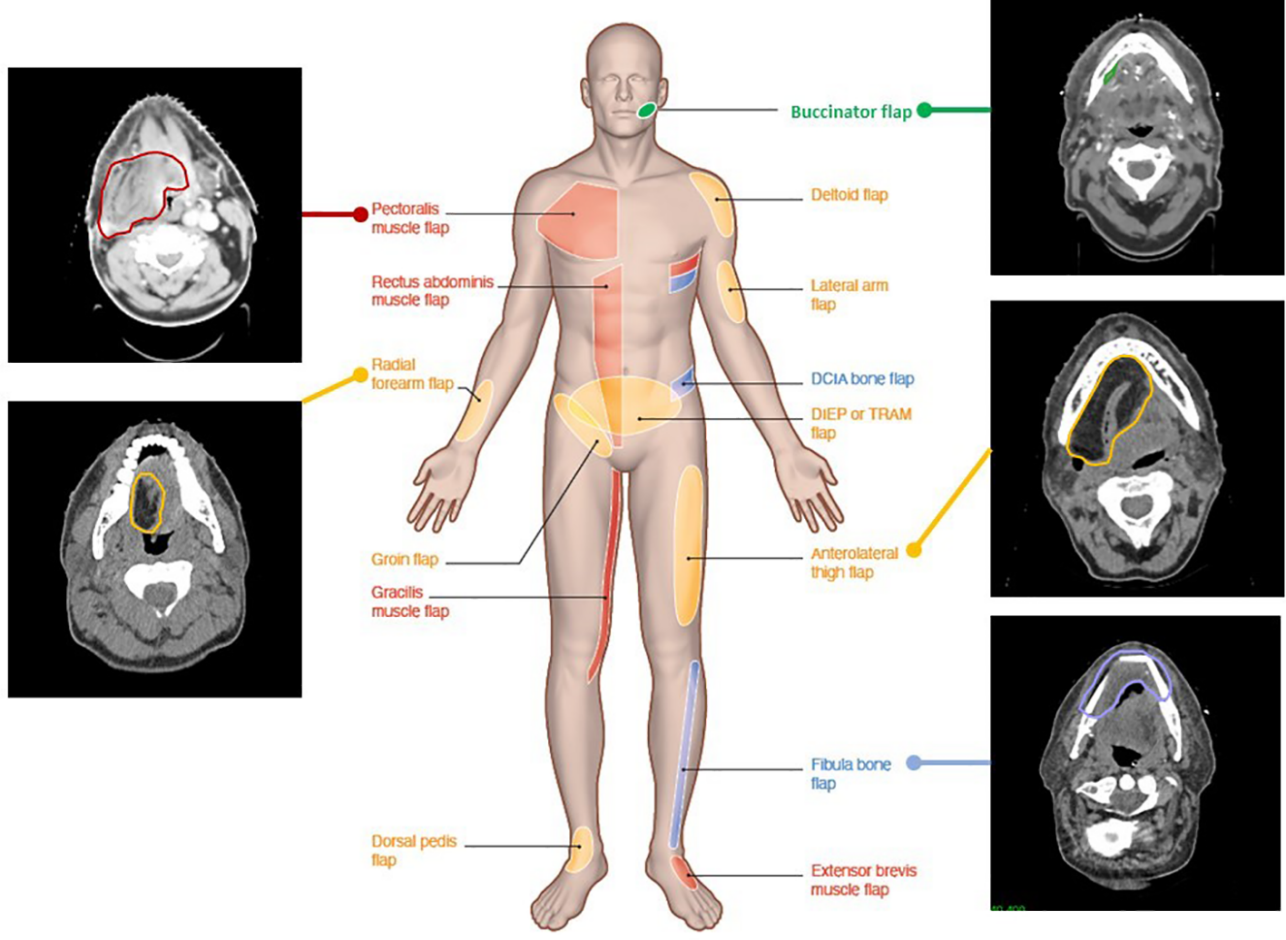
Computed tomography images illustrating various flap reconstructions in head and neck surgery. Flap donor sites from various donor body parts (middle male figure) and their aspect and contours on postoperative radiotherapy planning CT. Red, pedicled flaps; blue, bone free flaps; orange, soft tissue free flaps; green, local flaps.

**Figure 2** illustrates the CT aspect of five common flaps. Myocutaneous (muscle, skin, and fat), fasciocutaneous (fascia, skin, and fat), mucosal (mucosa), visceral (viscera and fat), or bone (bone and soft tissues) flap components exhibit distinct features on imaging (45, 46). On CT, the fatty portion of a flap is hypodense in the [-140; -40] Hounsfield unit (HU) range. On MRI, fat tissues have a short relaxation time and T1/T2 hypersignal, which can hide tumor enhancement in T1 or be mistaken for edematous hypersignal in T2, suggesting the use of fat signal suppression (STIR) gadolinium enhancement T1-weighted sequences. With time, fat can show atrophy or slight augmentation/edema. On CT, the muscular flap component is usually striated, isodense to native muscle and relatively flat. On MR, its signal and enhancement pattern is moderate to intense (15, 47, 48). In the early postoperative period, the flap muscle may be edematous, T1-hypointense, T2-hyperintense, and diffusely T1 gadolinium-enhanced. Later, denervated muscles (such as in myocutaneous flaps) are susceptible to volume loss and fatty replacement of muscle (15, 47, 49, 50), with heterogeneous T1/T2 signal and CT hypodensity. The skin usually exhibits enhancement in fasciocutaneous flaps. These are less susceptible to atrophy (being without muscle) but more to skin fibrosis. Mucosa in flaps usually presents like native mucosa. Local mucosal (such as buccinator) and visceral (such as jejunum) flaps exhibit normally enhanced mucosa, with mesenteric fat carrying donor vessels and lymphatic nodes on CT. Some bone flaps may be distinguished from the native bone by shape and a higher corticated portion. Baseline postoperative CT assessment at 3 months should be proposed for later recurrence assessment (46).

### 4.2 Spontaneous flap changes over time

Various changes that are specific to flaps and distinct from native tissues may occur.

#### 4.2.1 Flap failure

Patient factors, such as age and comorbidities (including diabetes mellitus and malnutrition), tumor site, red blood cell transfusion, wound healing and infectious complications, operating room duration, length of hospital stay, or risk of early postoperative readmission are important predictors of flap failure (51, 52). On one hand, flaps, being autologous tissues, are not susceptible to auto-immune rejection. On the other hand, recent isolated reports suggest that anti-cancer immunotherapy might compromise flap vitality (53), possibly due to a higher risk of thrombosis (54, 55).

Flap failure, defined as partial flap or total flap loss, is rare overall. The ultimate success rates of micro-anastomosed free flaps are about 85%–90% (56), but they refer to the final surgical outcome, which may have necessitated flap repair or replacement for early flap complications, which vary from 30% to 80% across surgical series (15, 57). The incidence of perioperative free flap compromise is low, with successful salvage in up to 70%. When the flap is again compromised, second salvage has been reported to be successful in 30% of free flaps (79 in 3,510 flaps in 2000–2020) (58).

Complications must be detected early because of the narrow window of opportunity for flap salvage. Early flap failure is often detected clinically by the loss of viability of a small superficial portion of the soft tissue flap (palor, color, temperature, etc.). Nevertheless, more in-depth (at the tissue–flap junction) complications may be missed by clinical observation. Delayed flap failure may also occur. It can be depicted on CT as intra- or peri-flap fluid collection >4 cm, intra- or peri-flap air collection >2 cm, and fistula to the adjacent aerodigestive tract or skin (59). Early free flap failure may primarily be due to veinous thrombosis (usually within 72 h). Arterial thrombosis can occur even earlier and can be severe (60). Prolonged ischemia time during surgery and revision of microvascular anastomosis are risk factors for flap failure (61, 62). Early thrombectomy, if feasible (short ischemia), leads to better survival than vein revision to neighboring branch veins (58). Local and pedicle flaps can also suffer vascular damage by vessel stretch or torsion. Finally, early flap loss due to necrosis is unlikely due to radiation-induced damage of vascular anastomosis or thrombosis (7).

Imaging is rarely prescribed for the suspicion of early flap failure as clinical suspicion may suffice. Early MRI might, however, be useful to assess deeper tissue damage (63).

Doppler monitoring, hyperspectral imaging (HSI), and microdialysis are gaining popularity as promising techniques for monitoring flap viability. Doppler monitoring is a minimally invasive method where a probe is situated near the vascular anastomosis, while HSI evaluates soft tissue parameters like temperature, re-capillarization time, and flap turgor (64), and microdialysis can be utilized for soft tissue or bone (65). Thiem et al. incorporated HSI as a complementary tool to traditional clinical evaluations for assessing free flap viability in reconstructive surgery (64). Their non-randomized clinical study, which included 54 primary and 11 secondary reconstructions, found that HSI detected perfusion compromise notably earlier than clinical assessments alone, leading to re-exploration decisions about 4.8 ± 5 h sooner (*p* < 0.001). With a flap salvage rate of 63%, the study implies HSI as a potentially valuable addition to postoperative flap monitoring procedures.

#### 4.2.2 Atrophy

In flaps with a significant muscle component, motor nerve section is usually preferred to its preservation to avoid inadequate contractions. Loss of muscular activity usually results in fatty conversion and overall flap volume reduction of >40% 2 years after surgery (50, 66, 67). Flap volume reduction is related to its components (67, 68) and weight loss, while age is associated with less skin retraction (possibly due to less collagen) and atrophy (muscle/fat proportion). Muscle volume may decrease to less than a third of its immediate postoperative volume (50), while fat volume may be more stable (50, 68), with <20% volume reduction (39, 69). Fat volume reduction in flaps seems to correlate with poorer outcomes: fat volume has been shown to decrease by 70% in patients dying of their HNC and increase to >115% in patients free of disease (50). Fatty tissue atrophy might also be related with flap innervation, direct tissue damage, ischemia during surgery (39), or patency of the flap pedicle and anastomotic blood flow (50, 70). Motor innervation might contribute to preventing atrophy.

#### 4.2.3 Fibrosis

Fibrosis refers to a dynamic, pathological process of parenchymal cell damage, stromal remodeling, and tissue contraction, particularly in the muscle component (71). It manifests as a delayed and progressive reduction in tissue elasticity and flexibility, associated with poorer function. Severe fibrosis can manifest as stiffened cervical skin, dense platysma, and flattened internal jugular vein, which becomes anterior to the common carotid artery.

Ultrasound, CT perfusion (blood flow, blood volume, and time maximum intensity projection), and functional MR [dynamic contrast-enhanced with fractional plasma volume, volume transfer constant, peak enhancement, and time to maximum enhancement and imaging diffusion-weighted imaging (DWI)] can also help to differentiate tumor recurrence from postoperative changes and fibrosis. Soft tissue density on CT and iso-hypointensity on T1/2 sequences represent a scar or fibrosis.

#### 4.2.4 Other flap changes

Other changes include wound dehiscence (separation of skin edges 30%), native skin breakdown, and the presence of a pharyngocutaneous fistula or the conditions requiring re-operation such as venous congestion and ecchymosis, seroma or hematoma, infection (56, 63, 72). Heterotopic ossifications occur from osteogenesis from the vascularized periosteum. They are depicted as linear periosteal attenuations of the vascular pedicle on CT, i.e., ossified vessels. They are reported in up to 50% of patients with fibula flaps within 1 month to 2 years postoperatively (73, 74). Spontaneous bone resorption may occur by about 0.2 mm/year in the native mandible (edentulous being at a higher risk) and transplanted bone to a lesser degree (75). Bone flaps might be at a higher risk for osteoradionecrosis than native bone. Fixation screws and plates at the flap–native bone interface can be responsible for metallic artifacts (with image blurring) and backscatter radiation, which might increase their susceptibility to osteoradionecrosis (76) (see Section 5).

### 4.3 Tumor recurrence after reconstructive flap surgery

#### 4.3.1 Dissemination pathways in the presence of flaps

Some local myomucosal flaps might, in theory, harbor microscopic disease according to the concept of field cancerization. However, this phenomenon seems to be exceptional (77). Excluding local flaps, flaps are non-tumoral tissues that should not contain tumor cells, in contrast to native tissues surrounding the resection area. Therefore, this junction area between residual native tissues post-resection and the flap would be the area where recurrence would more likely occur before colonizing the flap by contiguity.

Permeation through the flap skin directly has been reported (unpublished) but might indeed have been initiated centripetally from lateral surface mucosal margins in tongue carcinomas, for example. Other unusual patterns of spread have been anecdotally reported where tumor disseminates from the native tissue–flap junction along flap muscle fibers (72).

Apart from these anecdotical situations, this junction area between residual native tissues post-resection and the flap should be identified for careful assessment during follow-up. Based on preclinical or translational studies, flap composition (fat, fascia, smooth or striated muscle, and bone or deperiosted bone) might influence the patterns of tumor recurrence. Tissue transfer and integration into a different site on the same body relies on complex epithelial remodeling between the macroscopic or microscopic components of flaps and surrounding potentially tumorigenic native tissues. It is unknown whether typical “anatomic barriers”, such as fasciae, may retain a barrier effect in flaps. It is also unknown whether adipose tissue transfer from flaps could turn into cancer-activated adipocytes and whether controversies on lipotransfer apply (78). Similarly, it is unknown whether fibroblasts could turn into cancer-activated fibroblasts in their new environment. Anecdotical reports indicate that such interactions might be worth investigating (79). It is also possible that clotting and inflammatory processes contribute to tumor dissemination and local immunotolerance. However, free flaps allow a wider resection, and their use may be associated with fewer marginal resections and better oncologic outcomes by decreasing local recurrences (13, 14, 80, 81). Similarly, poRT studies suggest that relapses occur mainly at the junction between the flap and the native tissues (see chapter 5).

#### 4.3.2 Imaging of recurrence

Imaging reveals sharp boundaries between the flap and normal tissues, indicative of benignity, while poorly limited contrast-enhanced edges of the surgical field may signal local tumor recurrence (14, 72, 81–84). CT characterizes recurrence as a slightly hyperattenuating contrast-enhanced infiltrative mass or nodule, with radionecrosis and infection being the primary differential diagnoses. The less accessible deep flap–tissue junction, especially areas of weakness like muscular perimysium and bone, necessitates a careful inspection.

In a retrospective study, Kim et al. analyzed 93 HNC patients, 82% of whom had a previous surgery (85). Among them, 79 (84.9%) patients had recurrent tumor at the primary site confirmed *via* surgical resection. The study’s strength lies in using histological evaluation of the neck specimen as the reference standard for both true-positive and true-negative findings. The authors reported the sensitivity and specificity of MRI to be 0.75 and 0.99, respectively, and for PET/CT to be 0.86 and 0.95.

MRI and PET-CT are typically prescribed when relapse is suspected. It is worth noting that flap enhancement is not specific for recurrence, varying from no to diffuse enhancement with neither T2 hypersignal nor contrast-enhanced T1 being specific for flap failure (47). MRI protocols should include 3DT1, axial fat-saturated T2, post-contrast fat-saturated T1, and diffusion-weighted (DWI) sequences. Low apparent diffusion coefficient (ADC) on DWI-MRI could be indicative (47, 86), but it is not specific for small foci of viable cancer cells along flap muscle fibers.

18FDG PET/CT improves sensitivity and specificity in recurrence detection. Ravanelli et al. evaluated [18F] FDG PET-CT’s usefulness in following up surgically treated oral tongue squamous cell carcinoma (87). Among the 87 included patients, 68 (78%) had a flap. The study classified PET uptakes in the oral cavity as functional, suspicious, or highly suggestive of neoplastic recurrence. FDG uptake was observed in the oral cavity during follow-up in 59 (68%) patients, primarily deep in the floor of the mouth, near the interface between the native tissue and the flap. This was explained as compensatory hyperactivation of the contralateral extrinsic oral muscles, which may retract the flap in distorted and asymmetric anatomies. Nodular regions of FDG uptake deep to the flap at the native flap–tissue junction warrant particular attention (88).

Finally, CT is the preferred initial imaging modality for recurrence detection, while PET-CT and MRI may detect early relapses, considering the false-positive rates of PET-CT for up to 12 weeks (88). The use of MRI to quantify flap changes and correlate them with functional outcomes is worth investigating (69, 86).

## 5 Effects of postoperative radiotherapy on flaps

### 5.1 Efficacy and toxicity of postoperative radiotherapy

Radiation-associated soft tissue injury can range from acute reversible toxicities to destructive degenerative processes such as osteoradionecrosis, which can contribute to flap failure and delay oral rehabilitation. The surgical literature suggests that postoperative radiotherapy (poRT) alters the functional outcomes of reconstructive flap surgery (89–91). Brachytherapy increases the rate of complications in patients undergoing microvascular free tissue transfer (92). With external beam radiotherapy, there is a clear imbalance between patients receiving surgery alone or poRT in patients with poor prognostic factors. Most studies have been small, non-randomized, and retrospective without propensity score matching (**Table 2**).

**Table 2:** Studies on postoperative radiotherapy effects in patients undergoing flap surgery.

Name	Type	Location	Number of patients	Number of poRT+ patients	Flap	Median total dose poRT+ (Gy)	FU (months)	Toxicity > 2
Chang et al., 2022 (128)	Retrospective	Hypopharynx (78%)	36 (14 surgery alone including 7 recurrences)	22 (61%) (4 recurrences)	Free jejunal flap	NA	46	Swallowing impairment (2/22) early complication (leakage, hematoma, flap failure) 3 vs. 2 late complication (PEG insertion due to swallowing difficulty, focal stenosis) 2 vs. 2 QLQHN35 by Cronbach’s alpha coefficient 3 and 12mo 0.88 identical between poRT+ and poRT-
Lee et al., 2022 (103)	Retrospective	Oral cavity/oropharynx (67%)	87	87	Various flaps	60–65	4	Initiation of poRT increased for flap complications in 5.7% of patients
Yamazaki et al., 2021 (127)	Retrospective	Oral cavity	35	16	Free forearm flap (77%)	60–70	NA	Mean flap volume reduction at 12 months = 80% (CT), no difference between poRT+ and surgery alone but authors reported shrinkage in cutaneous flaps with RT, not in myocutaneous flaps
Gérard et al., 2020 (112)	Retrospective	Oral cavity/oropharynx (76%)	100 poRT+ (surgery without a flap 46, flap 54)	54 poRT+ flap+	Various flaps	60–70	39	No poRT- group, more acute and late toxicities (esophageal stenosis, swallowing difficulties) with a flap (vs. without) (full flap inclusions in high-risk CTV; larger volumes irradiated)
Lilja et al., 2018 (126)	Prospective	Oral cavity/oropharynx (93%)	88 (5 prior RT)	39 poRT+ (89%)	Radial forearm (77%)	NA	12	Quality of life and sensitive assessment, anosmia (2/34), no comparative analysis of poRT+ vs. poRT-
Haymerle et al., 2018 (125)	Retrospective	Oropharynx	13	10 poRT+	Free forearm flap	55	26	Mean flap volume (CT) reduction at 3mo 56% poRT+ 3 poRT-, 12 months 70% poRT+ 21% poRT-
Mohamed et al., 2017 (80)	Retrospective	Oral cavity	54 recurrences after poRT+ (out of 289 patients)	54 poRT+	NA	60	NA	Insufficient reporting of flap-related outcomes; one flap recurrence
Tarsitano et al., 2016 (89)	Retrospective	Oral cavity (tongue)		11	Anterolateral thigh free flap	62	12	Mean flap volume reduction after 12 months = 44.2% (MR based analysis)
Bitterman et al., 2015 (120)	Prospective	Oral cavity, oropharynx	15 (9 poRT+, 4 RT post flap, 2 poRT-)	15	Various	NA	3	31.3% and 39.2% similar volume reduction at 1 year after surgery
Higgins et al., 2012 (124)	Retrospective	Parotid		13	Anterolateral thigh free flap	55	22	Mean flap volume reduction after 6 months = 8.12% (CT-based analysis)
Shin et al., 2012 (90)	Retrospective	Oral cavity (tongue)	31	13	Free forearm flap	61.5	43	Tongue mobility Impairment (10/13) similar with poRT-/poRT+, swallowing capacity superior poRT- vs. poRT+
Airoldi et al., 2011 (123)	Retrospective/consecutive patients	Oral cavity	153	36	Free forearm flap	61.3	54	No comparative poRT- group, dysphagia 53%
Choi et al., 2004 (91)	Retrospective	Oral cavity, oropharynx	100 (28 poRT-, 37 prior RT, 35 poRT+)	35	Fibula flap	64	11	Similar complication rates—any severity 54% poRT-, 65% prior RT, 46% poRT+

poRT, postoperative radiotherapy; RT, radiotherapy; NA, not available; CT scan.

Surgically treated HNSCC patients undergo poRT based on the presence/absence of pathological risk factors for recurrence (93, 94). These risk factors include positive surgical margins and extranodal extension, lymphovascular invasion, perineural invasion (PNI), advanced T stage (pT3–4), high grade, advanced N stage (N2–3), involved nodal levels IV–V (NCCN), worst invasion pattern (WPOI), metastatic nodal burden, and ratio in neck dissection specimen (95–100). In patients with high risk factors such as extranodal extension (ENE) or positive surgical margins (PSM), post-operative radiation therapy (poRT) enhances loco-regional control, disease-free survival, and overall survival. Moreover, when concurrent cisplatin chemotherapy is implemented, the 5-year survival rates see an improvement from 40% to 50% (95). poRT is considered in intermediate-risk patients with pT3–4 disease or positive nodes without ENE or PSM (101). However, the benefit of poRT is less clear when the sole adverse factors present are perineural invasion (PNI), lymphovascular invasion, differentiation, or specific patterns of invasion (93). In a randomized trial updated for long-term outcomes, there was no dose–response above 63 Gy (102). The time interval between surgery and poRT has more impact on survival than dose and should be ≤6 weeks (NCCN), and flaps do not seem to compromise it (103). In high-risk patients, the primary tumor bed with PSM/ENE receives 60–66 Gy in 1.8–2-Gy fractions, and the areas at risk for microscopic involvement receive 50–54 Gy. Most evidence for poRT using modern RT techniques are retrospective, and adherence to guidelines is low in practice. In patients with HPV+ oropharyngeal cancer, minimally morbid surgery +/- poRT (21%–58% of cases) or chemo-poRT (16%–62% of cases) is often proposed (104). Deescalated poRT dose/volumes are being investigated in trials to achieve better long-term functional outcomes in patients with HPV+ oropharyngeal cancer (105). In the poRT with IMRT era (poIMRT), the patterns of failure are mostly in-field (≈65%) or marginal (≈25%). Marginal relapses in the presence of ENE appear to require generous margins around the tumor bed (≈1.5 cm) (106–108); some occurred at the match line at the field junction or near the spared parotids in steep dose gradients (109). Out-of-field relapses have been reported mostly as contralateral nodal relapses in pN2a-b oral cavity cases and seem unlikely to be salvaged (110, 111). In oral cavity cancers, most (55%) local failures appeared to occur in the central high-dose region and less frequently in central intermediate-low-dose regions (18.5%) and out-of-field-dose regions (16.7%) (80). Local patterns of failure after poIMRT are less reported.

Considerable gaps in knowledge exist on how to handle a flap when poRT is needed and the transfer of flap-related advances to poRT (7, 45, 104, 112–114). Flaps now represent a substantial proportion of patients operated on for mucosal HNC (11). The patterns of relapse have only been anecdotally reported, with relapses that occur being mostly marginal and not intra-flap (115). Flap reconstruction anticipation before poRT seems to improve the margin quality and oncologic outcomes (80).

### 5.2 Postoperative radiotherapy planning in the presence of flaps

#### 5.2.1 Planning CT protocols

At the time of poRT planning, the operative bed often shows a dramatically modified anatomy [such as edema, fluid/air bubble collection (hematoma, lymphocele), thickening of the skin, and platysma or reticulation of the subcutaneous fat] secondary to tissue loss, deformation, and inflammatory changes of adjacent normal structures and, increasingly often, the presence of reconstructive tissues (flaps) and materials. The post-reconstruction imaging interpretation is challenging to define the clinical target volume of the operative bed. Moreover, not all radiotherapy departments perform optimal CT due to logistics constraints (lack of radiologists for customized protocols, lack of physicians to monitor allergic contra-indications to iodine agents, and longer acquisition times with contrast enhancement). Due to its benefit on delineation accuracy (116), contrast-enhanced poRT planning CT may be recommended using a split-bolus technique to visualize arteries, veins, native tissues, residual tumors, and flaps. The intravenous iodine agent is infused at 2.5 mL/s, with 55 mL injected first and another 55 mL after a 40-s delay. Thin-section (≈1 mm) image acquisition is performed after a 90-s delay, followed by multiplanar reconstructions. Image artefact blurring is often reduced using MAR algorithms but still warrants inaccurate manual overriding for dosimetry. Multi-energy/spectral CT is largely implanted in radiodiagnostics but less systematically in radiotherapy departments. Moreover, proprietary treatment planning system (TPS) formats often alter spatial resolution to limit computational times, resulting in blurred/indistinct images hampering flap visualization. Knowing flap-related findings on high-resolution imaging is critical to distinguish flaps from surrounding anatomy and assess their spontaneous changes over time and after poRT on the one hand and tumor recurrence or flap complications on the other hand (117).

#### 5.2.2 Flap delineation

PoRT recommendations lack guidelines on flap delineation and dose constraints, a situation possibly attributed to the complexity and time-consuming nature of delineation, given factors such as heterogeneity, altered anatomy, and interpretation of operative and pathology reports (104, 114, 118, 119). However, an accurate definition of flap boundaries is critical for successful flap sparing (120).

The current approach often encompasses over 80% of flaps in target volumes, typically focusing on the hypodense fatty portion of the flap (112). Dose–volume effects, unfortunately, have been scarcely documented (112). To address this, automatic segmentation tools utilizing neural networks may facilitate a more systematic flap delineation. Moreover, virtual marking of resection borders using a navigation pointer and titanium ligature clips can aid in delineation, as suggested by a prospective study (120). Establishing a standardized approach to flap delineation could significantly assist in defining flap dose–volume effects (see Section 5.3).

**Figure 3:**
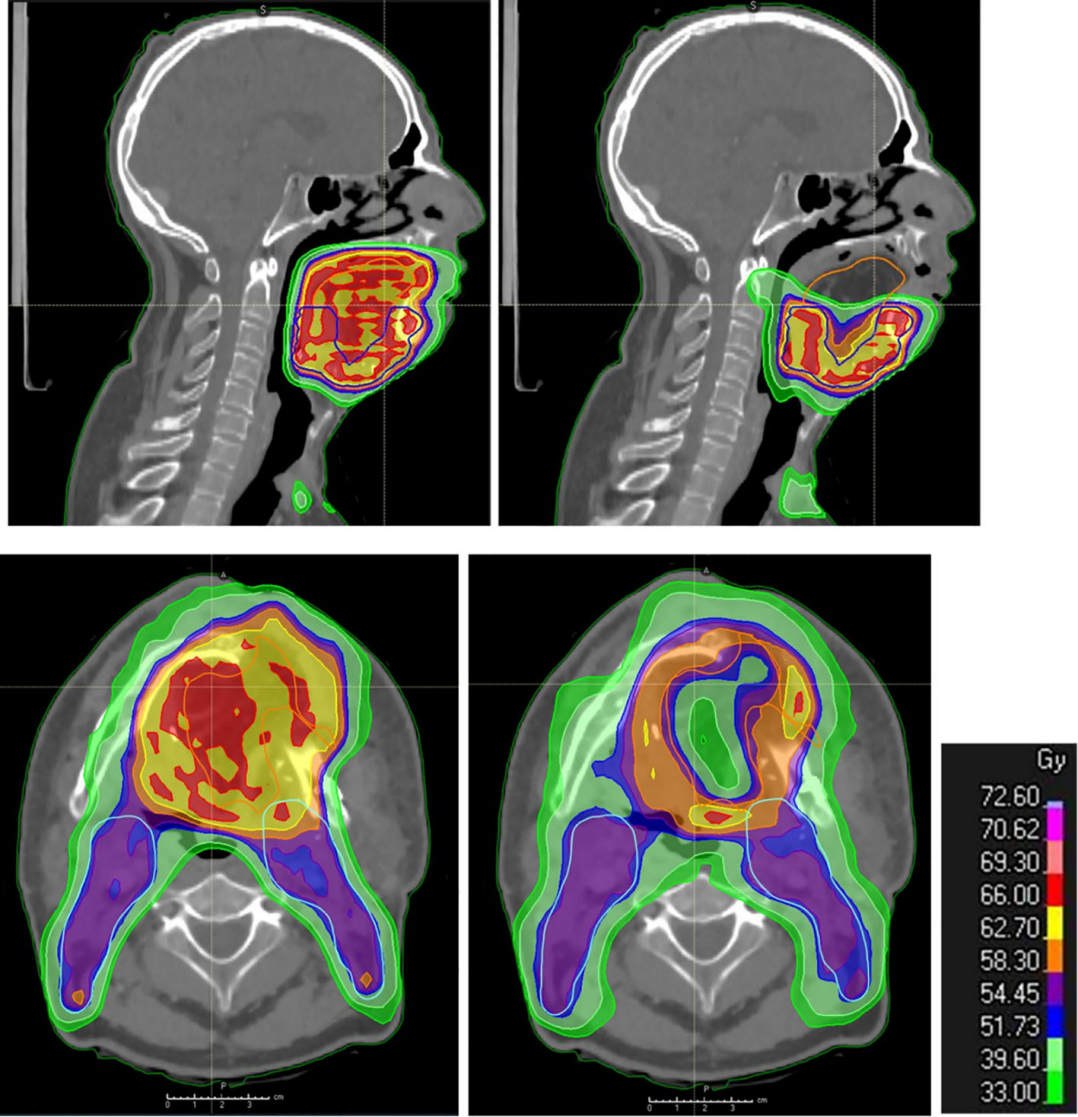
Post-operative radiation therapy planning in the presence of a flap. Dosimetry showing standard coverage (left: upper panel in sagittal view and lower panel in axial view) and flap sparing (right: upper panel in sagittal view and lower panel in axial view; the cranial part of the flap is spared as well as the central part, while the flap–native tissue junction receives full tumor dose) in flap (yellow contour) areas distant from the native tissues at risk of harboring microscopic tumor in a patient with reconstructive anterolateral thigh flap surgery for oral cavity cancer.

With the use of IMRT, high dose conformity in at-risk areas can be achieved while preserving other areas through a technique known as dose painting. By utilizing dose painting by contours (DPBC), the incidence of late mucosal ulcers can be reduced, thereby enhancing the patients’ quality of life (121). This protocol could be adapted for flap sparing, maintaining the dose at the native tissue–flap junction and limiting the dose to 40 Gy for portions of the flap distant from this junction, such as the center and surface (122)—for example, in a reconstructed mobile tongue, only the deep and lateral junction and an area of 6 mm into the flap at risk may receive an intermediate or high dose of 60–66 Gy, guided by the pathological report (**Figure 3**). Longitudinal studies are required to evaluate if this strategy leads to improvements in morphology, volume, texture, and functional outcomes without raising the risk of local recurrence.

The identification of flaps as organs at risk and their accurate delineation would allow for specific sparing strategies. This would not only optimize the radiation dose but also minimize flap-related complications (Section 5.3), improving the overall patient outcomes. Future advancements may include the use of AI for precision delineation, but that remains a promising area for exploration.

### 5.3 Contribution of poRT to flap changes or complications

A total of 10 key articles primarily addressed radiation-induced changes or complications in the context of flap-based head and neck cancer (HNC) radiotherapy (**Supplementary Data S2**) (89, 90, 103, 112, 123–128). The reports indicated a significant mean flap reduction ranging from 8% to 70%.

#### 5.3.1 Radiation-induced atrophy

**Figure 4:**
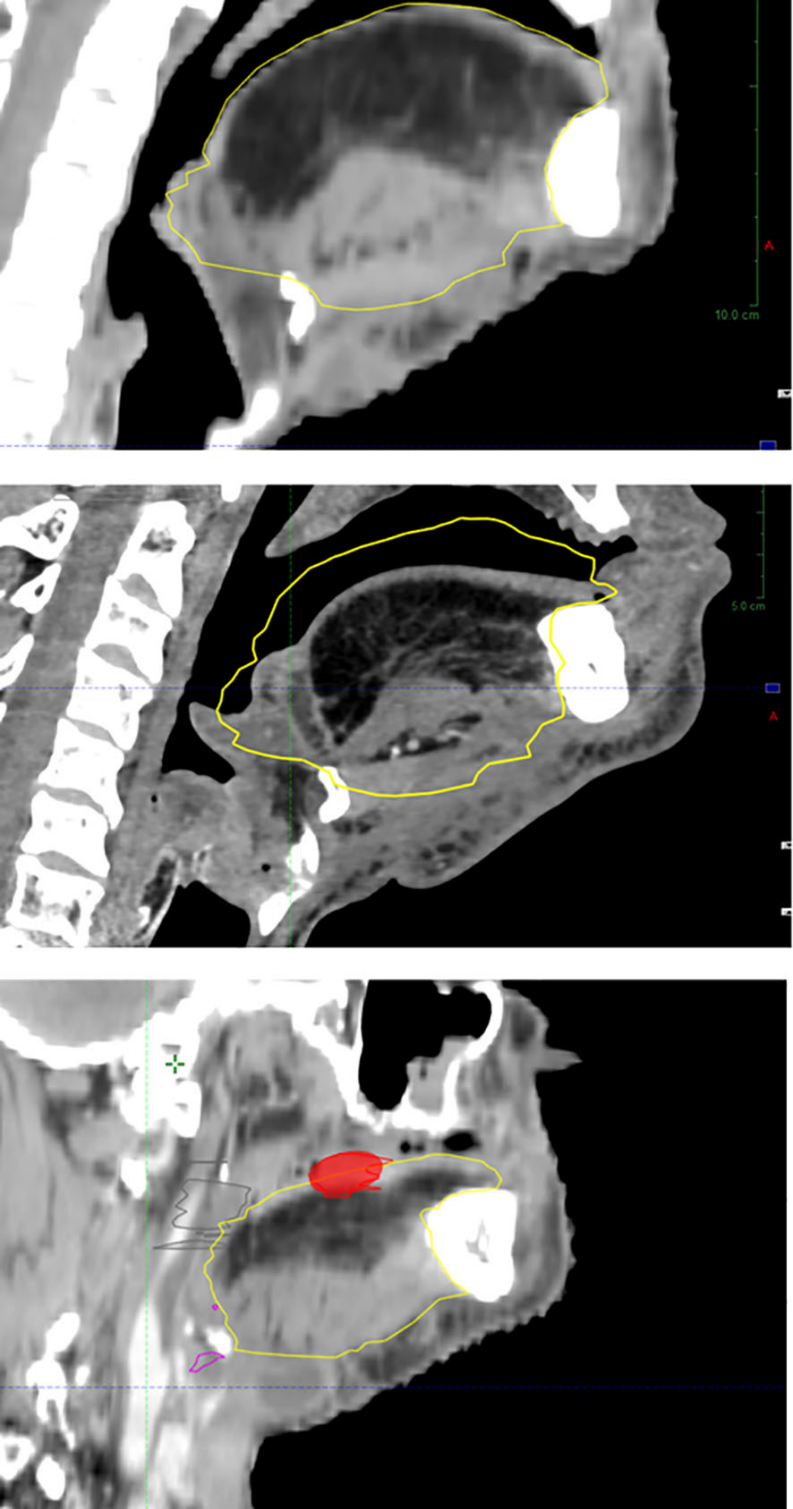
Flap atrophy over time with local relapse at the flap–native tissue junction (baseline, upper panel; atrophy at 1 year, intermediate panel; relapse at 2 years, lower panel).

In addition to spontaneous and host-dependent atrophy (50), poRT further contributes to flap volume reduction (68, 129) (**Figure 4**). With an average flap volume loss of 34%, 39% was reported in patients with poRT *versus* 31% in patients without poRT (130), probably by muscle atrophy, which itself is related to innervation (37, 39). This occurred despite efficient flap vascularization possibly through radiation endarteritis and chronic ischemia (39). PoIMRT may be offered to reduce high-dose irradiation to some parts of the flap (37).

#### 5.3.2 Radiation-induced fibrosis

Radiation-induced fibrosis (RIF) is depicted clinically as a woody aspect (131, 132). Mild to moderate RIF is frequent (133). A vascularized fibrotic scar depicts low soft tissue T2 signal (lower than recurrence) and ill-defined margins and enhancement and might be difficult to differentiate from recurrence (134). Along with fibrosis, RIF might participate in altered soft tissue flap versatility (7). It may be more severe in flaps compared to other tissues (37, 39); it often accompanies subcutaneous fibrosis and mucosal edema (130).

#### 5.3.3 Osteoradionecrosis

Osteoradionecrosis is a known complication following definitive IMRT, manifesting in 1%–10% of patients typically within 1 to 2 years post-treatment. In the context of reconstructed bone, osteoradionecrosis rates have reached up to 34% following reconstructive flap surgery and poRT (135). The potential risk factors include the number of osteotomies, which can lead to impaired vascularization due to the increased dissection of periosteum from the bone and decreased resistance to radiation-induced vascular damage, the exposure of hardware or plates and the potential for bacterial seeding, and the use of bone free flaps (in place of the native mandible), which are particularly susceptible as the bony margins have only undergone the initial stage of healing before being exposed to poRT. The presence of dental implants might also contribute to osteoradionecrosis. Therefore, in anticipation of poRT, the decision to perform a large number of osteotomies should be carefully balanced against the requirements of achieving a highly conformal reconstructed bone and maintaining long-term viability. The soft tissue provided must be sufficiently broad to allow perfect coverage of the osteosynthesis plates, even if it means harvesting a second soft free flap (136). Besides this, caution should be exerted with radiation fields passing through metals, especially for patients treated with proton therapy. Dose backscatter from plates and screws might result in increased dose to the native and flap segments at their junction, but lack of data prevents risk estimates (137). Above all, a poRT dose over 60 Gy (possibly through pericapillary fibrosis) is associated with an increased risk of developing osteoradionecrosis in the bony flap, along with large resection, large RT field, and chemotherapy (135). This issue must be considered, especially when reoperation for secondary bone reconstruction and dental rehabilitation is required after poRT. It is worth noting that dose escalation does not provide survival benefit (102, 138) but induces significant collateral tissue damage and acute and late toxicity (135), suggested to not exceed 66 Gy in the poRT setting in the absence of macroscopic tumor.

#### 5.3.4 Other radiation-induced effects

PoRT may result in acute and delayed injury to the skin, including color, texture, and elasticity, increased enhancement and thickening of flap components such as skin and platysma, fat reticulation, and reduced vessel lumen (15). Facial nerve management (dissected when macroscopically involved and reconstructed using the hypoglossal nerve, for example) has been extensively described in parotid tumors to restore facial palsy. poRT did not compromise functional outcomes, suggesting that flap reinnervation is feasible before poRT if needed (139).

#### 5.3.5 Impact of postoperative radiotherapy on flap failure

There is a persistent ambiguity surrounding the risks associated with prior RT and poRT. Long-term radiation-induced adverse effects are well documented, including alterations in smooth muscle density, endothelial cell dehiscence, vessel wall fibrosis, connective tissue scarring, and numerous vascular lesions exhibiting microthrombi and heightened proadhesive and prothrombotic properties (140). In the short term, small local flaps can typically attain autonomy from their pedicle within 3 weeks (up to 12 weeks for larger free flaps) through neovascularization by adjacent recipient tissues. As poRT should be administered within 42 days, by which time the vascular supply has usually been consolidated, there is scant evidence to suggest that poRT could jeopardize the flap’s vascular supply in the short term. Consequently, early flap loss might be more attributable to the surgical procedure rather than poRT. Nonetheless, poRT can independently induce an increased expression of cytokines and leukocyte adhesion molecules and promote clotting *via* mechanisms such as leukocyte or platelet endothelial adherence, thrombus formation, a sustained increase in plasminogen activator inhibitor PAI-1, persistent inflammation due to NF-κB, and atherosclerosis (141, 142). Hence, there is ongoing debate regarding the immediate effects of poRT on the incidence and severity of flap complications (122). The need for prospective controlled studies to elucidate this matter is pressing.

#### 5.3.6 Impact of postoperative radiotherapy on functional flap outcomes

Functional deleterious impact of poRT on flaps occurs by volume reduction, muscle fibrosis, subcutaneous tissues, and skin retraction (39). However, prospective data showed that some patients recovered, while others did not (143). Interestingly, functional kinetics were highly correlated with survival. Tissue effects and their reversibility are less understood. Therefore, imaging and functional data on poRT/no poRT series are needed to correlate flap changes with outcomes and investigate the causality and responsibility of poRT. PoRT may delay sensory recovery, while motor recovery does not seem to be altered if one considers facial nerve outcomes in irradiated parotid tumors. Further data are, however, warranted (34).

## 6 Conclusion and perspectives

Reconstructive flap surgery in HNC patients represents a significant interdisciplinary challenge that facilitates the personalization of multimodal treatment de-escalation. The present review has some limitations. It is not systematic or quantitative, and only 10 studies specifically focusing on flap and poRT were found and meticulously analyzed in this review. Despite this, the narrative approach allowed us to cover a broader spectrum of information than a systematic review could. The poRT planning process could be improved by integrating interdisciplinary definitions of spread patterns and standardizing multimodal imaging for flap delineation both prior to and during the follow-up stages of poRT (144–147). Moreover, the evaluation of functional flap outcomes should be incorporated into the treatment process, and dedicated trials are needed to optimize patient care.

## Author contributions

JT: Writing – original draft, Writing – review & editing. FC: Writing – original draft, Writing – review & editing. AB: Writing – original draft, Writing – review & editing. SD: Writing – original draft, Writing – review & editing. P-YM: Writing – original draft, Writing – review & editing. AM: Writing – original draft, Writing – review & editing. CD: Writing – original draft, Writing – review & editing. BD: Writing – original draft, Writing – review & editing.
