# Supplementary material for: Reconstructive flap surgery in head and neck cancer patients: an interdisciplinary view of the challenges encountered by radiation oncologists in postoperative radiotherapy
Source: Front Oncol. 2024 Apr 11;14:1379861. doi: 10.3389/fonc.2024.1379861 (PMC11043495; doi:10.3389/fonc.2024.1379861)
Supplement: Supplementary file 1 [file DataSheet_1.docx]

**Sup Data 1:** Flow-chart


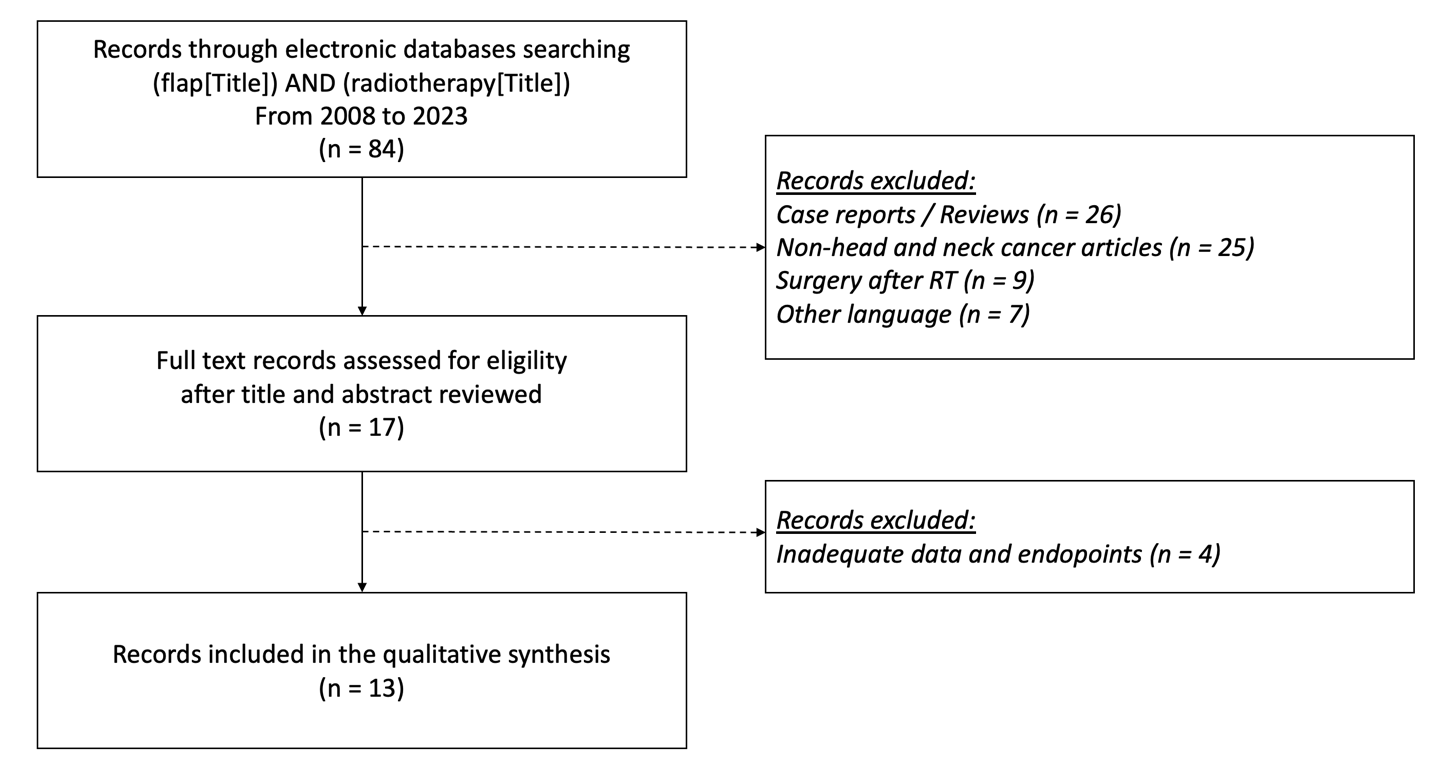


**Sup Data 2:** Recent Key Studies on Postoperative Radiotherapy Toxicity in Patients Undergoing Flap Surgery

| Name | Type | Location | Number of patients with poRT | Flap | Median Total Dose (Gy) | FU (months) | Toxicity > 2 |
| --- | --- | --- | --- | --- | --- | --- | --- |
| Chang et al. 2022 | Retrospective | Hypopharynx (78%) | 22 (61%) | Free jejunal flap | NA | 46 | Swallowing impairment (2/22) |
| Lee et al 2022 | Retrospective | Oral cavity/Oropharynx (67%) | 87 | Various | 60 - 65 | 4 | Not associated with the delay between surgery and PORT |
| Yamazaki et al. 2021 | Retrospective | Oral cavity | 35 | Free forearm flap (77%) | 60 - 70 | NA | Mean Flap volume reduction after 12 months = 30% (CT based analysis) |
| Gérard et al. 2020 | Retrospective | Oral cavity/Oropharynx (76%) | 54 | Various | 60 - 70 | 39 | Fibrosis (10/54) |
| Lilja et al. 2018 | Prospective | Oral cavity/Oropharynx (93%) | 34 (77%) | Radial forearm (77%) | NA | 12 | Anosmia (2/34) |
| Haymerle et al. 2018 | Retrospective | Oropharynx | 13 | Free forearm flap | 55 | 26 | Mean Flap volume reduction after 12 months = 70% (CT based analysis) |
| Tarsitano et al. 2016 | Retrospective | Oral Cavity (tongue) | 11 | Anterolateral thigh free flap | 62 | 12 | Mean Flap volume reduction after 12 months = 44.2% (MR based analysis) |
| Higgins et al. 2012 | Retrospective | Parotid | 13 | Anterolateral thigh free flap | 55 | 22 | Mean Flap volume reduction after 6 months = 8.12% (CT based analysis) |
| Shin et al. 2012 | Retrospective | Oral Cavity (tongue) | 13 | Free forearm flap | 61.5 | 43 | Tongue mobility Impairment (10/13) |
| Airoldi et al. 2011 | Retrospective | Oral Cavity | 36 | Free forearm flap | 61.3 | 54 | Dysphagia (8/36) |
